# Similar degenerative patterns of olfactory receptor genes in the giant panda and marine carnivores

**DOI:** 10.1016/j.isci.2026.117936

**Published:** 2026-10-17

**Authors:** Liwen Kang, Junjie Chen, Mingsheng Hong, Wei Wei, Zuofu Xiang, Zejun Zhang

**Affiliations:** 1 College of Forestry, Central South University of Forestry and Technology, Changsha, Hunan 410004, China; 2 Key Laboratory of Southwest China Wildlife Resources Conservation (Ministry of Education), China West Normal University, Nanchong 637000, China; 3 College of Pharmacy, Chengdu University of Traditional Chinese Medicine, Chengdu 611137, China

**Keywords:** olfactory receptor genes, carnivora, gene loss, giant panda, ecological adaptation

## Abstract

Olfactory receptor (OR) genes constitute the largest mammalian gene family and are critical for olfactory perception and ecological adaptation. OR gene variation is associated with interspecific differences in habitat, diet, and sensory reliance, yet the relative contributions of these ecological factors to OR evolution remain unclear. Here, we systematically analyzed OR repertoires across 25 carnivoran species occupying diverse niches. Marine carnivorans exhibit convergent OR degeneration, with fewer intact genes and ancestral orthologs, higher pseudogene ratios, and relaxed purifying selection compared with terrestrial species. Notably, bamboo-specialized giant pandas display identical degenerative signatures, possessing the smallest intact OR repertoire and most lost ancestral OR groups among terrestrial carnivorans. Our models show habitat primarily shapes intact OR quantity, while dietary specialization governs pseudogenization and selective constraints. These findings reveal convergent OR functional decay under aquatic adaptation and extreme dietary specialization, illustrating complex links between lifestyle and chemosensory genome evolution in mammals.

## Highlights

• Convergent OR gene degeneration in marine carnivorans and bamboo-specialized giant pandas
• Habitat primarily shapes intact olfactory receptor gene quantity across carnivoran species
• Dietary specialization dominates pseudogenization levels and selective constraint of OR genes

## Graphical abstract

**Figure:**
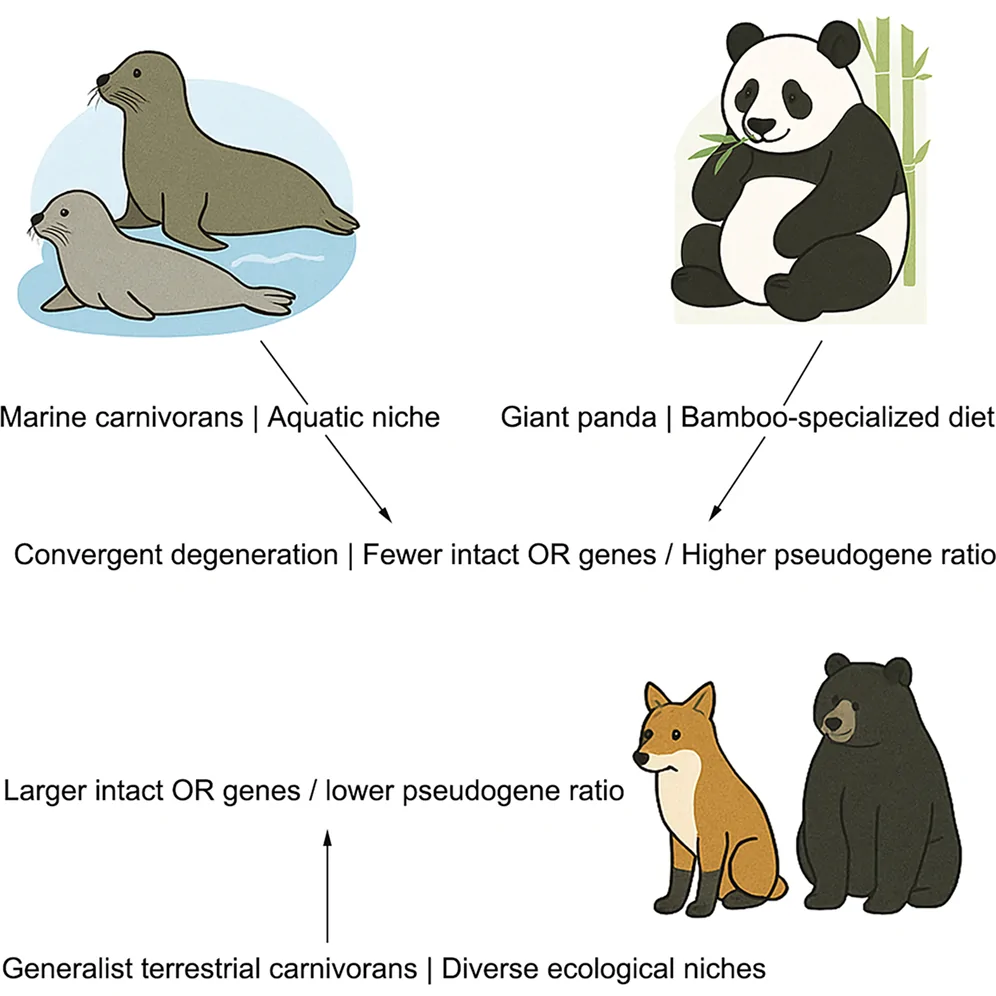

## Teaser

Zoology; Evolutionary biology; Phylogenetics

## Introduction

Olfaction is one of the most important sensory modalities in animals, playing a crucial role in perceiving the environment, locating food, selecting mates, and avoiding predators.^1^^,^^2^^,^^3^^,^^4^ Odorant molecules in the environment are detected by olfactory receptors (ORs) expressed in the olfactory epithelium of the nasal cavity.^5^^,^^6^^,^^7^ Intact OR genes are G protein-coupled receptors (GPCRs) characterized by seven α-helical transmembrane domains, forming a protein of approximately 310 amino acids.^8^ OR genes are widely distributed across chordates, spanning seven evolutionary clades including lancelets, jawless fish, jawed fish, amphibians, reptiles, birds, and mammals, and were first identified in rodents in 1991.^9^ Each OR gene encodes a receptor protein, enabling animals to discriminate among a wide variety of chemical compounds.^10^ A single odorant molecule can be recognized by multiple ORs, while a single OR can recognize multiple odorants. The combinatorial recognition of odorants by different ORs ultimately generates a unique neuronal odor-coding pattern, thereby allowing the identification of a vast array of environmental odors.^11^^,^^12^ Based on amino acid sequence divergence, OR proteins are generally classified into Class I and Class II, with the former preferentially binding water-soluble odorants^12^ and the latter binding hydrophobic odorants.^13^^,^^14^

The number of OR genes varies among mammals, and the OR gene repertoires of different species differ greatly,^12^ which may partly relate to differences in ecological niches.^3^ The most pronounced differences occur between terrestrial and aquatic mammals.^15^ For example, the African elephant possesses the largest known repertoire of putatively functional OR genes, encoding nearly 2,000 genes,^16^ whereas cetaceans harbor only a few dozen functional OR genes.^15^ During the transition from land to aquatic habitats, marine mammals underwent substantial functional OR gene loss; notably, toothed whales further evolved echolocation as an alternative sensory adaptation to the aquatic environment.^17^ Similarly, as a mammal, the platypus possesses approximately 350 functional OR genes, a number substantially lower than that of typical terrestrial mammals such as mice (∼1,000), dogs (∼811), and elephants (∼1,841), and even lower than its monotreme relative, the echidna (∼600). This reduced OR repertoire likely reflects the evolutionary shift from olfactory-based to electroreception-based prey detection associated with its semi-aquatic lifestyle.^15^^,^^18^ Primates also experienced accelerated OR gene loss during evolution, which was linked to the reduced reliance on olfaction associated with two major adaptive shifts: the acquisition of acute vision (including the fovea and loss of the tapetum) in the haplorhine ancestor, and the dietary transition from fruit foraging to leaf foraging in colobines, where odor cues are less critical for food selection.^19^^,^^20^ Humans, chimpanzees, orangutans, rhesus macaques, and marmosets retain only about 300–400 functional OR genes,^21^ fewer than most non-primate terrestrial mammals.

Liu et al.^15^ annotated OR genes from 23 mammalian species. Terrestrial species included opossums, African elephants, giant pandas and others, whereas marine mammals covered bowhead whales, minke whales, Weddell seals, and so on. Jian et al.^22^ identified OR genes of giant pandas and polar bears. In 2023, Zhou et al.^23^ performed OR gene annotation on six Ursidae species of Carnivora: giant panda, spectacled bear, American black bear, brown bear, polar bear, and Asiatic black bear. Notable discrepancies in the number of intact OR genes and the proportion of pseudogenes were detected for the giant panda, a species shared across all three studies. Liu et al.^15^ reported 611 functional OR genes in giant pandas, Jian et al.^22^ recorded 645 intact OR genes, and Zhou et al.^23^ documented only 408 functional OR genes for this species.

Multiple comparative genomic studies across mammals have confirmed that aquatic adaptation leads to convergent degeneration of olfactory receptor genes.^15^^,^^24^ Nevertheless, their studies only included a small number of carnivoran species and failed to systematically resolve the evolutionary patterns of OR genes within Carnivora. Existing OR-related studies on Ursidae are mostly restricted to partial taxa and lack systematic comparisons across the entire order Carnivora, making it difficult to clarify the divergent genomic characteristics of olfactory receptors between terrestrial species with specialized diets and marine carnivorans.

Carnivoran species exhibit interspecific divergence in habitat and dietary strategies, and previous studies have documented degenerative signatures including extensive OR gene loss and elevated pseudogene ratios in marine carnivorans. Accordingly, this study proposes the central hypothesis: habitat, degree of dietary specialization, and habitat breadth are key factors that potentially drive the divergent evolution of the OR gene family in Carnivora. Based on this hypothesis, we put forward the following predictions: compared with generalist terrestrial carnivorans with broad diets and wide niches, marine carnivorans and giant pandas—species with specialized diets and narrow ecological niches—possess significantly fewer intact functional OR genes and a higher proportion of pseudogenes.

To empirically test these hypotheses and predictions, we annotated, analyzed, and compared a taxonomically balanced sample of 25 carnivoran genomes to gain insight into the underlying evolutionary patterns.

## Results

### Overall identification of OR genes in carnivoras

Based on protein sequence similarity and homology, we identified a total of 20,685 intact OR genes from the whole-genome sequences of 25 carnivoran mammals. Detailed information for these results is shown in Figure 1A and Table S1. We found that marine carnivores possessed significantly fewer OR genes (333–367) compared to their terrestrial relatives (702–1,393) (Figure 1B; Mann-Whitney *U* test, *p* < 0.0005). Meanwhile, the proportion of OR pseudogenes was significantly higher in marine carnivores (49.31%–59.82%) than in terrestrial carnivores (24.15%–49.75%) (Figure 1C; Mann-Whitney *U* test, *p* < 0.0005). Notably, the giant panda exhibited the lowest number of OR genes (702) among terrestrial carnivores, considerably fewer than other ursid species (813–1,276), and its OR pseudogene proportion was exceptionally high (49.75%), reaching levels comparable to marine carnivores (49.31%–59.82%). As shown in Figure 1D, no significant correlation was observed between the number of OR pseudogenes and the number of intact OR genes per genome (*R*^2^ = 0.1277; *p* > 0.05). The proportion of OR pseudogenes was negatively correlated with the number of intact OR genes (Figure 1E; *R*^2^ = 0.5015; *p* < 0.0005).

**Figure 1:**
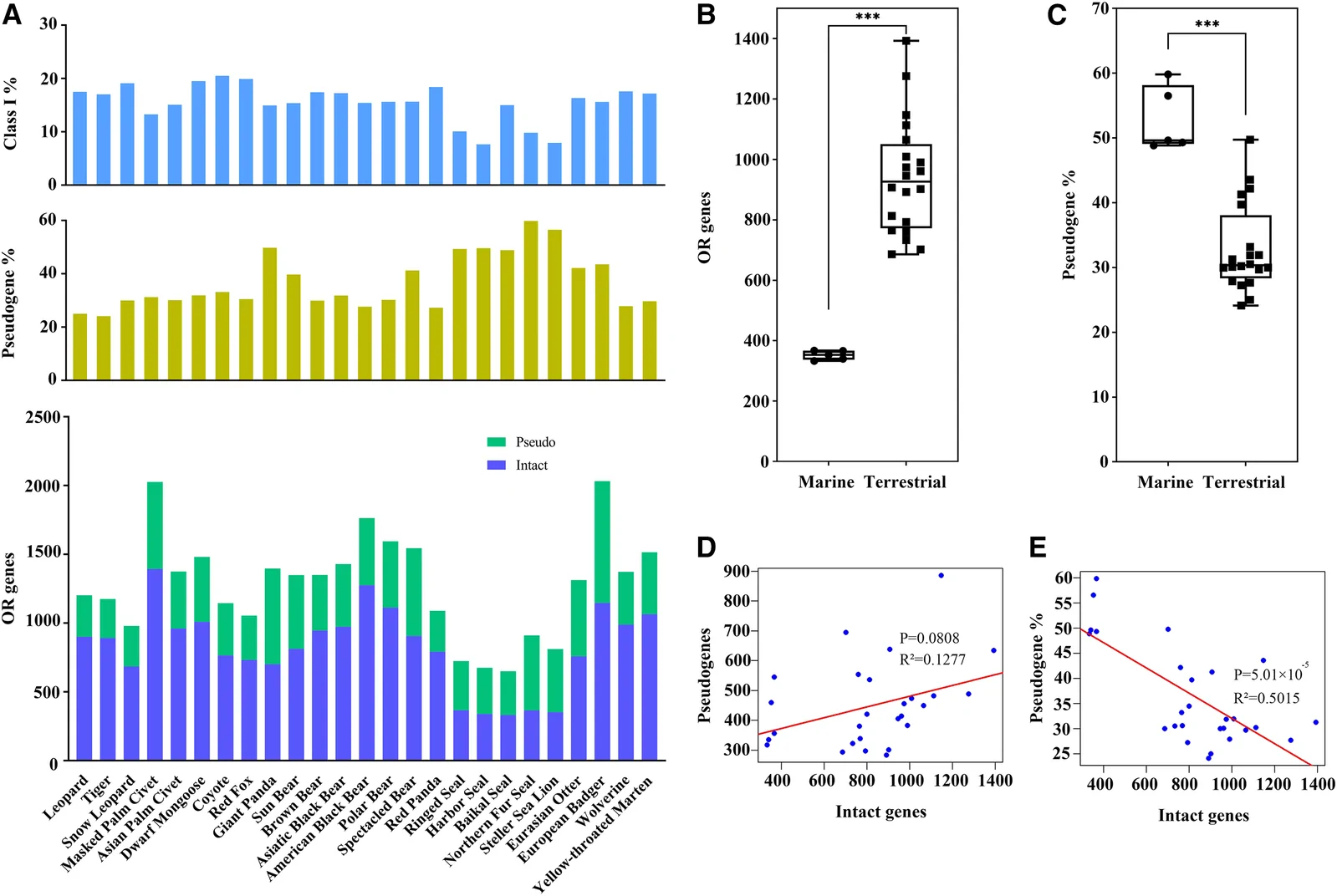
Comparison of OR gene repertoires in carnivoran genomes (A) Proportion of Class I OR genes (calculated as the number of intact Class I genes divided by the total number of intact OR genes) and pseudogene proportion (calculated as the number of pseudogenes divided by the total number of OR genes). (B) Comparison of intact OR gene numbers between marine and terrestrial carnivorans (Mann-Whitney *U* test, *p* < 0.0005). (C) Comparison of OR pseudogene proportions between marine and terrestrial carnivorans (Mann-Whitney *U* test, *p* < 0.0005). (D) No significant correlation was observed between the absolute number of OR pseudogenes and intact OR gene numbers across genomes. (E) OR pseudogene proportion was significantly negatively correlated with intact OR gene numbers.

### Orthologous gene groups (OGGs)

We identified a total of 374 orthologous gene groups (OGGs) that contained two or more intact OR genes, together with 75 singleton genes, from our 25 carnivoran species. Based on sequence similarity with intact OR genes, all pseudogenes were also assigned to these 374 OGGs (see STAR Methods for details). As shown in Figures 2A and 2B, most OGGs contain a relatively small number of both intact OR genes and pseudogenes. Among the 374 OGGs, the mean and median numbers of intact genes per OGG were 54.8 and 28.5, respectively, while the corresponding mean and median numbers of pseudogenes were 21.6 and 11. Across all OGGs, the number of intact OR genes was positively correlated with the number of pseudogenes (Figure 2C; *R*^2^ = 0.886, *p* < 0.0005), indicating that OGGs with more intact genes also tended to harbor more pseudogenes. Within the same OGG, although OR gene sequences are relatively conserved, the number of OR genes varies considerably among species. For example, in OGG2-1, the American black bear (124), spectacled bear (114), and masked palm civet (101) possess a large number of intact OR genes, whereas ringed seal (16), harbor seal (13), and baikal seal (14) have only a small number of intact OR genes.

**Figure 2:**
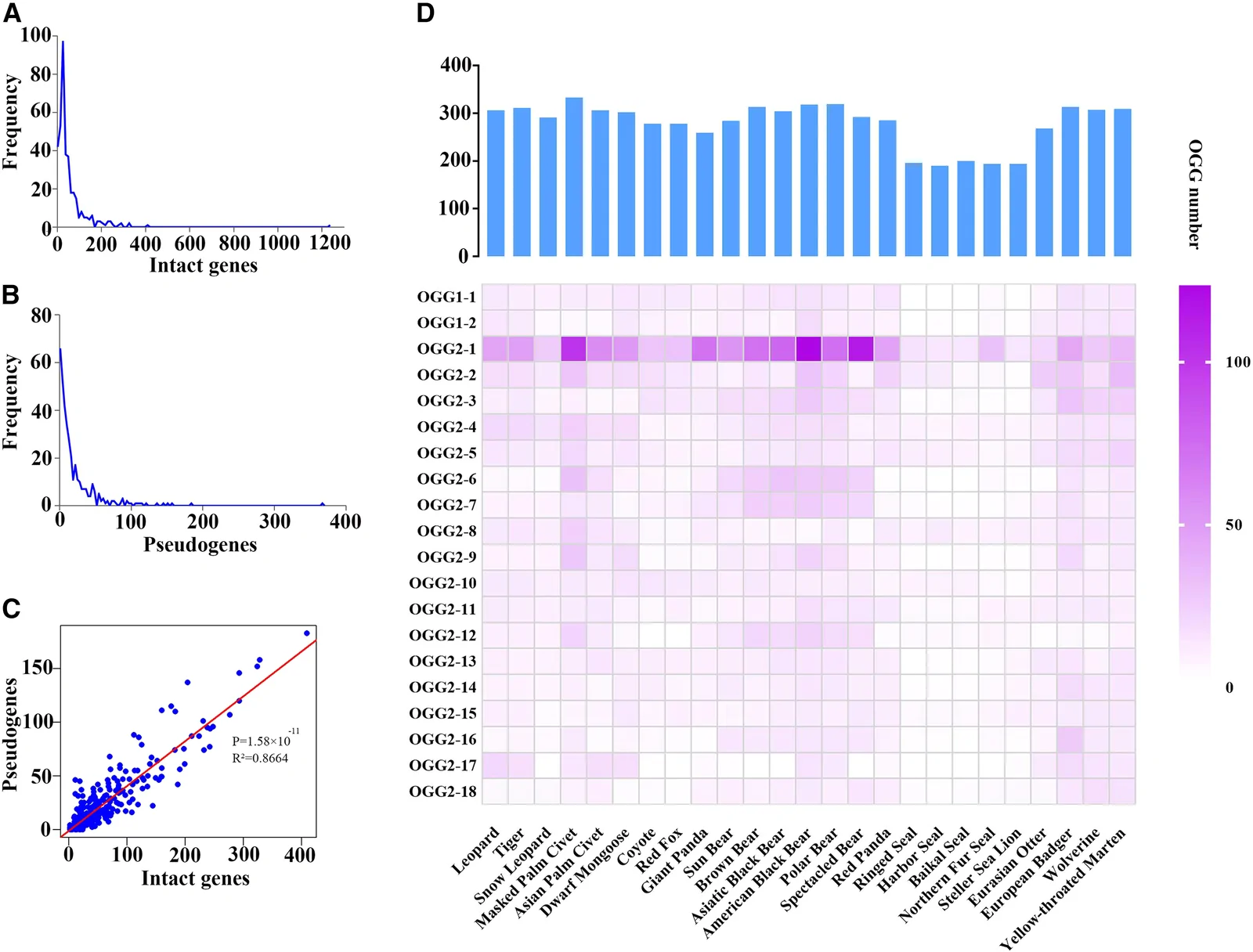
Comparison of gene numbers within OGGs (A) Distribution of intact OR genes within OGGs. (B) Distribution of OR pseudogenes within OGGs. (C) Positive correlation between the number of intact OR genes and OR pseudogenes across OGGs. (D) Distribution of OGG counts across species (upper image) and gene IDs of the 20 largest OGGs (lower image).

As shown in Figure 2D, we compared the number of OGGs among the 25 carnivore species. The number of OGGs varied substantially across species, ranging from 194 to 333. Marine carnivores (190–200) exhibited significantly fewer OGGs than terrestrial carnivores (259–333) (Mann-Whitney *U* test, *p* < 0.005). Among terrestrial carnivores, the giant panda had the fewest OGGs (259). Focusing on the 20 largest OGGs, we observed extensive species-specific duplications. For example, in the masked palm civet, OGG2-1, OGG2-2, and OGG2-6 each contained more than 30 members, whereas in the Asian black bear, OGG2-1 and OGG2-6 also had more than 30 members each (Figure 2D).

### Selective pressure

The ω values for all 25 carnivoran species were below 1, indicating that OR gene functions are under purifying selection. In the comparison of ω values between Class I and Class II genes, the former was found to be significantly lower than the latter (Figure 3B; *p* < 0.0005), indicating that class II genes are more evolutionarily dynamic. As shown in Figure 3C, OGGs containing genes from marine carnivores exhibited significantly higher ω values than those from terrestrial carnivores, indicating relaxed purifying selection in marine carnivores. Among terrestrial carnivores, the giant panda has the highest ω value (0.3). The majority of OGGs had ω values ranging from 0.2 to 0.4 (Figure 3D).

**Figure 3:**
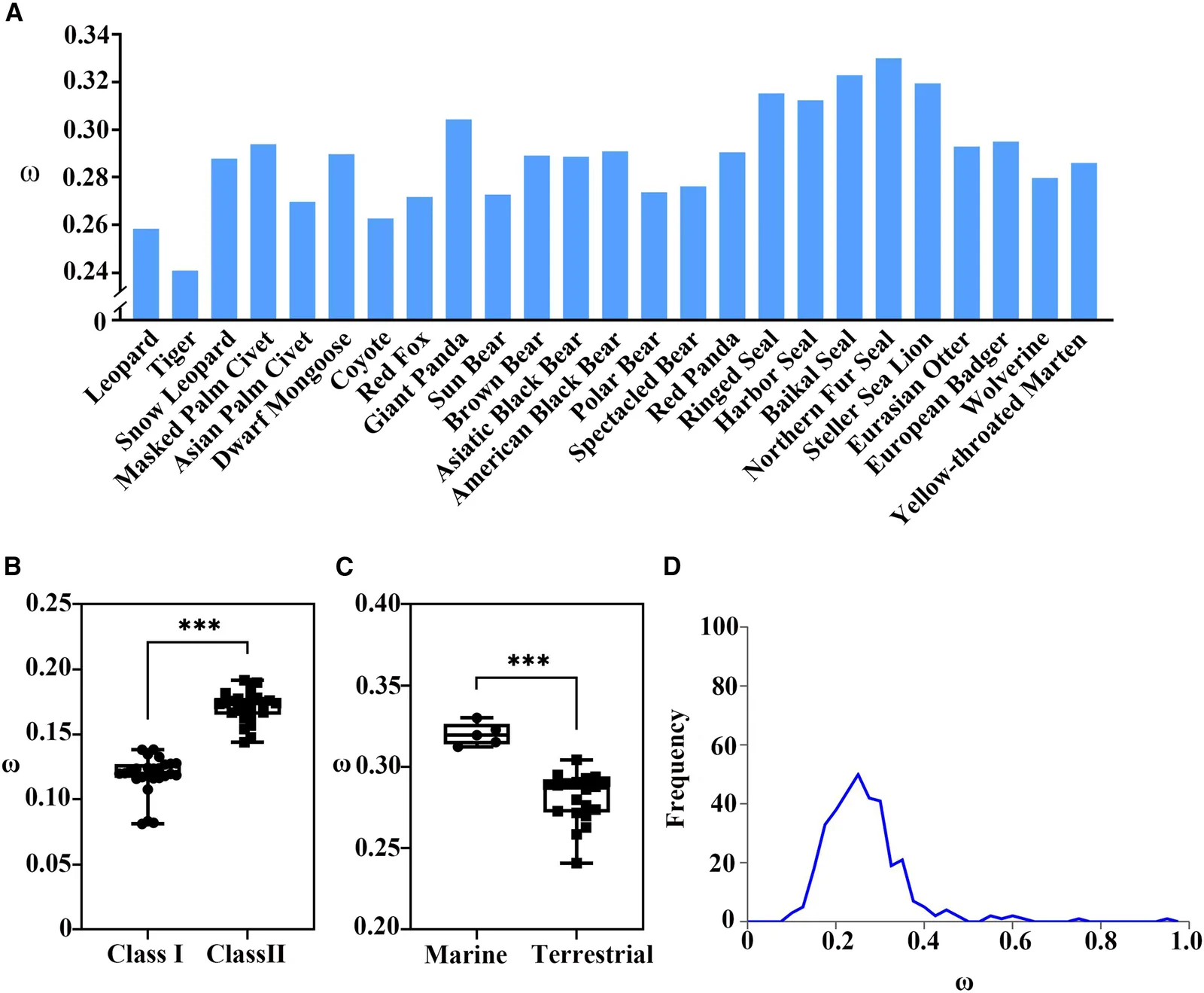
Comparative analysis of selection pressures on OR genes (A) Estimated ω values for OGGs across 25 carnivoran species. (B) Boxplot compares ω values between Class I and Class II OGGs. (Mann-Whitney *U* test, *p* < 0.0005). (C) Boxplot compares ω values of OGGs between marine and terrestrial carnivorans (Mann-Whitney *U* test, *p* < 0.0005). (D) Distribution of estimated ω values across OGGs.

### OR genes gains and losses

By definition, all genes within a single OGG are presumed to descend from one most recent common ancestral gene (MRCA). Nevertheless, orthology inference carries inherent uncertainty stemming from incomplete genome assemblies, fragmented OR sequences, and limits of clustering algorithms. Therefore, the 374 OGGs recovered in our analysis represent a computationally estimated ancestral repertoire rather than the definitive true number of ancestral intact OR genes in the carnivoran common ancestor. Due to gene gains and losses, these genes exhibit variation across different species. During the evolution of marine mammals and their closely related terrestrial relatives, we estimated the rates of OGG gain and loss for 374 OR genes along each branch. Extensive gains and losses occurred across different lineages (Figure 4). Consequently, even when two species have similar numbers of OGGs or genes, their OR repertoires may differ substantially. For instance, the northern sea lion and northern fur seal each possess 194 OGGs, but only 162 OGGs are shared between them. Furthermore, every one of the 25 mammalian species has lost dozens to more than one hundred of complete OR genes that were present in MRCA. Notably, marine carnivores lost far more ancestral OGGs (174–184) than terrestrial carnivorans (41–115) (Figure 4, Mann-Whitney *U* test, *p* < 0.0005). Among terrestrial carnivores, the giant panda lost the largest number of ancestral OGGs (115).

**Figure 4:**
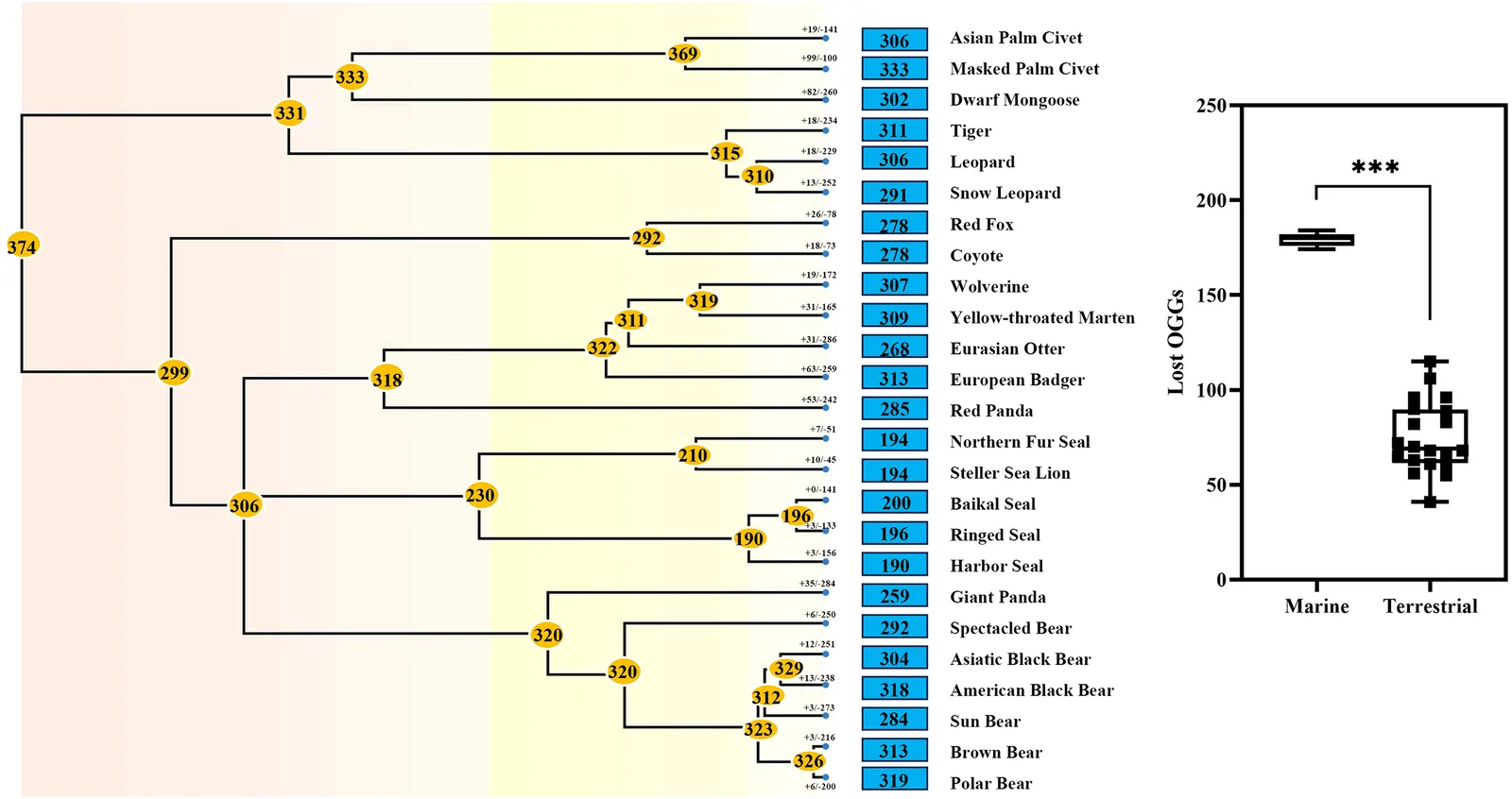
Changes in OGGs during carnivoran evolution and species ecological data Blue boxes indicate the number of OGGs with intact OR genes in extant species. Yellow ellipses indicate the number of intact OGGs inferred at ancestral nodes. Differences in OGG loss numbers between marine and terrestrial mammals are shown by boxplots.

### Ecological correlates of OR genes

To explore the regulatory effects of three ecological traits—habitat, diet, and habitat breadth—on the copy number of functional OR genes, pseudogene proportion, and evolutionary selection pressure (ω) in carnivorans, we constructed distribution-adapted generalized linear models (GLMs) and quantified the independent explanatory power of each predictor via deviance reduction.

For the count of intact functional OR genes, a negative binomial GLM with log link exhibited robust overall fit (null deviance = 155.79, residual deviance = 24.73, AIC = 320.28). Terrestrial and generalist species possessed significantly more intact OR genes (terrestrial: *β* = 0.581, *p* = 0.00195, with an average count 1.79 times that of aquatic species; generalist diet: *β* = 0.434, *p* = 0.01686, with an average count 1.54 times that of specialist species), whereas habitat breadth exerted no significant effect (*p* > 0.05). Deviance decomposition identified habitat type as the primary predictor of copy number variation, with explanatory power ranked as habitat > diet > habitat breadth (Figure 5A).

**Figure 5:**
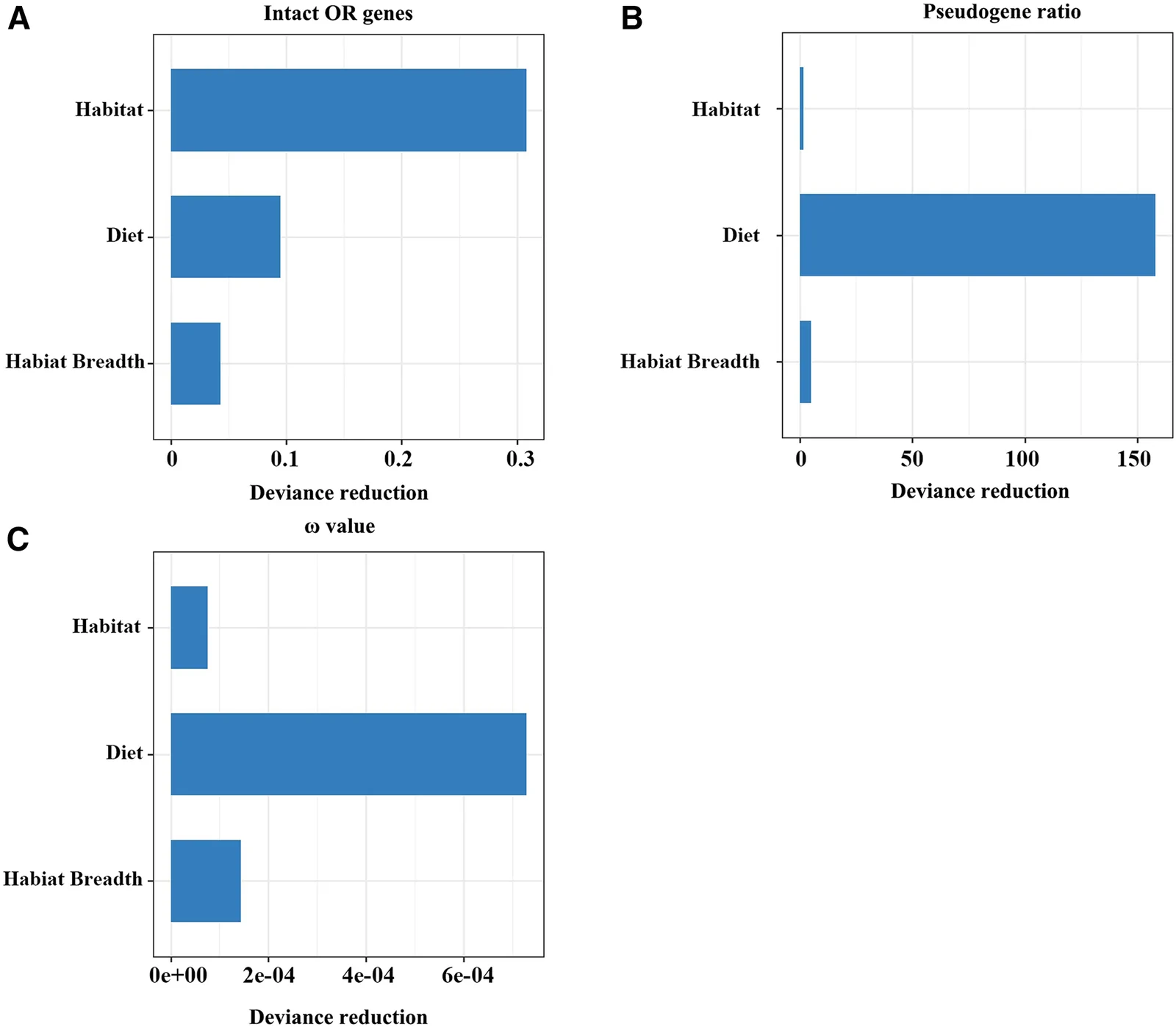
Bar plots show deviance reduction values derived from generalized linear models, which reflect the explanatory power of three ecological factors (Habitat, Diet and Habitat breadth) on key olfactory receptor evolutionary phenotypes across mammalian species (A) Total number of intact OR genes. (B) Proportion of OR pseudogenes. (C) Mean ω (dN/dS) values calculated from OR orthologous gene clusters.

For the proportion of OR pseudogenes, a binomial logit GLM showed strong model performance (null deviance = 1155.82, residual deviance = 415.55, AIC = 609.67). Generalist species had a markedly lower pseudogene proportion (*β* = −0.794, *p* < 2 × 10^−16^, with an odds ratio of only 45.2% relative to specialists), and habitat breadth was significantly positively correlated with pseudogene proportion (*β* = 0.020, *p* = 0.0257), while habitat type had no detectable influence. Diet type contributed far greater independent explanatory power than the other two predictors (Figure 5B).

For evolutionary selection pressure ω of OR genes, a Gaussian GLM revealed a marginally significant effect exclusively from diet type (*β* = −0.0311, *p* = 0.0577): generalist species exhibited lower ω values, indicating stronger purifying selection. Neither habitat type nor habitat breadth predicted ω significantly. Deviance decomposition confirmed diet type as the dominant factor driving divergence in ω (Figure 5C).

## Discussion

The OR gene family is one of the largest gene families in mammals, and both the number and functional status of OR genes vary significantly among different lineages, often closely associated with species-specific ecological adaptations.^3^^,^^24^^,^^25^ In this study, we systematically identified and compared OR genes from 25 carnivorans, including both marine and terrestrial species, to investigate the evolutionary patterns of the olfactory system under diverse ecological contexts and to explore the potential drivers of these patterns. One potential concern in comparative analyses of olfactory receptor gene repertoires is the accuracy of gene annotation across species. In this study, OR genes were identified using the Genome2OR pipeline, which has been shown to provide improved annotation performance compared with the widely used method described by Niimura.^26^ Comparison with the curated HORDE database further supported the reliability of our annotations, with 92.3%–99.4% of OR sequences identified by Genome2OR successfully mapped across species.^27^ Although differences in genome assembly quality may still influence pseudogene detection to some extent, these results suggest that the OR gene repertoires analyzed here are robustly annotated and suitable for comparative evolutionary analyses.

Notable numerical discrepancies in the count of functional intact OR genes of the giant panda have been reported across independent annotation studies, which stem from two core heterogeneous factors: distinct genome assembly versions and inconsistent OR identification pipelines. Jian et al.^22^ identified 645 intact ORs using an early fragmented scaffold-level giant panda genome and an in-house ORFAM pipeline with relaxed sequence filtering criteria, which retained numerous borderline candidate sequences. Liu et al.^15^ recovered 611 functional ORs based on low-contiguity genome assemblies via a simple tBLASTn homology search without multi-layer structural validation. In contrast, Zhou et al.^23^ adopted high-quality chromosome-level genome data and integrated a multi-step workflow combining tBLASTn alignment, Solar fragment assembly, GeneWise gene prediction, and HMMER conserved domain screening; their strict filtering standard yielded only 408 intact OR genes. In the present study, we implemented the Genome2or annotation pipeline and annotated 702 functional intact ORs for the giant panda. The larger number recovered in our work can be attributed to updated genome assembly resources, standardized full-length OR prediction algorithms, and optimized cutoff parameters that balance sensitivity and specificity compared to the three preceding studies.

We found that marine carnivores possess significantly fewer OR genes than terrestrial carnivores, along with a markedly higher proportion of OR pseudogenes. This result is consistent with previous studies on olfactory degradation in cetaceans and pinnipeds,^15^^,^^28^^,^^29^ suggesting that reduced reliance on olfaction in aquatic environments leads to functional losses in the OR gene repertoire. Notably, the highly dynamic evolutionary history of the OR gene family appears to be independent of genome organization and is more likely influenced by species-specific ecological niches.^3^

A previous study of 23 mammalian species revealed differences in OR gene repertoires between marine and terrestrial mammals. Although the giant panda was included in that study, which is the only species shared between their dataset and ours, no evidence of OR gene degeneration was reported.^15^ In contrast, our study focuses on carnivoran species and found that the giant panda exhibited the lowest number of OR genes among terrestrial carnivores in this study, while its proportion of pseudogenes is abnormally high, reaching the level observed in marine carnivores. This phenomenon suggests that the olfactory system of the giant panda may have undergone a similar degenerative process, with the driving factors being not an aquatic environment but rather its highly specialized diet (almost exclusively dependent on bamboo) and its forest-bamboo habitat.^30^^,^^31^ Similarly, higher primates generally possess fewer OR genes, likely due to their greater reliance on vision rather than olfaction.^21^ In non-human primates, folivory is significantly negatively correlated with the number of functional OR genes.^19^ Birds, which predominantly rely on vision, are generally less influenced by olfactory cues and possess markedly fewer intact OR genes than most mammals.^32^

OGG analysis indicated that approximately 374 ancestral OR genes were shared among the 25 carnivoran species examined, highlighting the conserved nature of this gene family. However, substantial variation in OR repertoire size across mammals was observed, largely driven by lineage-specific expansions in a few large OGGs (e.g., OGG2-1), which may be associated with unique ecological adaptations. Moreover, the fixation or loss of these new genes may reflect mammalian adaptations to their ecological niches. Deep-diving cetaceans have lost massive numbers of OR genes, retaining merely several dozen, while terrestrial mammals carry roughly three times as many OR genes as aquatic lineages. This disparity arises from two complementary evolutionary forces: gene duplication-driven expansion in terrestrial clades and extensive gene loss in aquatic clades.^3^^,^^15^ Despite the shared ancestral core, significant differences were detected among species in both OGG number and gene composition. For example, the masked palm civet exhibited massive gene expansions within certain OGGs, whereas marine carnivores and the giant panda showed reduced OGG numbers and extensive gene loss. These results suggest that the evolution of the OR gene family is shaped not only by large-scale gene losses but also by species-specific duplication events.^33^ This “conserved core plus lineage-specific diversification” pattern is consistent with findings in other large multigene families, such as the pancreatic ribonuclease (RNASE A) superfamily documented by Zhang.^34^ In leaf-eating colobine monkeys, a set of ancient, functionally essential RNase paralogs form the conserved core, while independent lineage-specific duplications of RNASE1 occurred in Asian and African leaf monkeys to adapt to folivorous diets via parallel amino acid substitutions. Additional mammalian gene families following the same evolutionary paradigm include visual opsins, bitter taste receptor (TAS2R) genes, and major histocompatibility complex (MHC) genes, all of which retain deeply conserved core domains while undergoing clade-specific copy-number expansion or contraction driven by ecological adaptation. The convergent loss of OR genes in marine carnivores likely reflects their unique aquatic sensory system, where most OR genes are under relaxed or absent selective constraints. Similarly, in the giant panda, extensive OR gene loss may be explained by the reduced diversity of odor cues in its “bamboo forest” environment, diminishing the evolutionary advantage of maintaining a large OR repertoire.^35^^,^^36^

It is well established that intact OR genes across all carnivorans are universally governed by purifying selection, as supported by the observation that all lineage-wide ω values remain below 1, meaning deleterious amino acid-altering mutations are still preferentially eliminated during evolution. Nevertheless, the intensity of purifying selection varies markedly across ecological groups, leading to distinct degrees of selective relaxation. Likelihood-based ω analyses revealed that Class II OR genes exhibited significantly higher ω values than Class I counterparts, indicating a more dynamic evolutionary trajectory driven by weaker baseline purifying constraints, which likely corresponds to their core function in detecting complex, variable environmental odorants.^37^ More importantly, orthologous gene groups (OGGs from marine carnivorans and the giant panda consistently displayed elevated ω values relative to other terrestrial carnivorans, demonstrating pronounced relaxation of purifying selection within these two lineages. This pattern reflects convergent attenuation of olfactory functional constraints rather than a complete loss of selective restriction: while mild purifying selection still operates to preserve partial receptor functionality, the strength of selection against disruptive mutations is substantially reduced. Such relaxed constraints accelerate the accumulation of loss-of-function variants and pseudogenization, consistent with diminished ecological reliance on olfactory cues under aquatic habitats or highly specialized bamboo-feeding niches.

Phylogenetic reconstruction indicates that the carnivoran common ancestor harbored a larger repertoire of intact OR genes, whereas each extant species has independently lost dozens to more than one hundred ancestral OR genes. The extent of gene loss is significantly greater in marine lineages compared to terrestrial ones, with the giant panda exhibiting the highest level of gene loss among terrestrial carnivores. These findings suggest that gene loss is the primary mechanism underlying the degeneration of the olfactory system, whereas lineage-specific gene expansions have maintained olfactory diversity and functional requirements in certain species.^15^

Our comparative genomic analysis of OR repertoires across 25 carnivoran species uncovered divergent evolutionary trajectories shaped by habitat and dietary niche: aquatic pinniped lineages exhibited severe contraction of functional OR genes with elevated pseudogenization and relaxed purifying selection, while terrestrial carnivorans maintained larger intact OR pools driven by recurrent lineage-specific duplications, with the bamboo-specialized giant panda showing a unique intermediate degenerative pattern distinct from other omnivorous bears. These findings align with a series of mammalian comparative OR studies focused on ecological adaptation. Zhou et al.^23^ systematically profiled OR gene compositions across six Ursidae species and demonstrated that the obligate herbivorous giant panda possesses a higher pseudogene ratio and has lost several plant odor-sensing OR subfamilies conserved in omnivorous bear species, consistent with our observation of weakened olfactory functional constraints in bamboo-feeding carnivorans. Parallel convergent OR degeneration across three independent marine mammalian clades (cetaceans, pinnipeds, sirenians) has been repeatedly validated; Liu et al.^15^ quantified drastically lower OR gain rates and drastically elevated gene loss rates in aquatic mammals relative to terrestrial counterparts, and further confirmed that OR orthologous groups under frequent birth-death turnover tend to evolve under weaker purifying selection, matching our ω-based selective pressure results across terrestrial and aquatic carnivorans. Beyond carnivorans, large-scale mammalian OR surveys^3^ established universal niche-associated differentiation: herbivorous mammals undergo massive expansion of OR 5/8/9 subfamilies linked to plant volatile detection, solitary terrestrial species accumulate more species-specific OR duplications than social taxa, while fully aquatic mammals converge on reduced functional repertoires regardless of phylogenetic origin. Such habitat- and diet-dependent remodeling of chemosensory gene families is not exclusive to mammals. In insects, divergent OR repertoire expansions and contractions tightly track host range, feeding habit, and diel activity; phytophagous insects evolve expanded OR clades for plant volatiles, while specialized parasitic lineages experience extensive OR pseudogenization,^38^^,^^39^ reflecting shared evolutionary logic where chemosensory gene turnover is tuned to ecological sensory demands, even across phyla with structurally distinct OR protein architectures. These results suggest that the reduction of OR genes in giant panda may be associated with certain ecological factors: 1) Giant pandas inhabit habitats overwhelmingly covered by bamboo, where diverse environmental odorants are less critical for survival, weakening the purifying selection maintaining full olfactory sensitivity; 2) Their nearly exclusive bamboo diet creates a stable, uniform food resource, further lowering the ecological demand for broad-spectrum odor detection, which lessens the selective pressure against disruptive mutations in OR sequences and accelerates pseudogenization.

### Limitations of the study

While our comparative genomic analysis reveals clear links between carnivoran ecological adaptation and the evolution of olfactory receptor (OR) repertoires, this work has some inherent limitations. First, our taxon sampling includes 25 carnivoran species. Although this dataset covers major carnivoran lineages and representative ecotypes, expanded sampling from additional taxa may help further refine the ecological associations we identified. Second, we annotated intact OR genes and pseudogenes based on available genome assemblies, and assembly quality and gene prediction artifacts may affect the estimation of OR repertoire size. Finally, our comparative analysis focuses on gene count variation rather than functional characterization of individual OR proteins, so we cannot directly link gene repertoire shifts to real olfactory perceptual differences in these carnivorans. Future functional assays and expanded taxon sampling will complement these genomic observations.

## Resource availability

### Lead contact

Further information and requests for resources should be directed to and will be fulfilled by the lead contact, LiWen Kang (liwenkang96@163.com).

### Materials availability

This study did not generate materials.

### Data and code availability

• Data: Annotated olfactory receptor sequences of 25 carnivoran species are provided in Table S4.
• Code: This paper does not report original code.
• Additional information: Any additional information required to reanalyze the data reported in this paper is available from the lead contact upon request, subject to their approval.

## Acknowledgments

This study was supported by the 10.13039/501100001809National Natural Science Foundation of China (grant no 32470516 and 31900337), the Giant Pandas International Cooperation Foundation of State Forestry Administration (2023), and the 10.13039/501100012166National Key Research and Development Program of China, grant no 2023YFF1305400. We also thank the anonymous reviewers for their constructive comments.

## Author contributions

L.K.: conceptualization, methodology, investigation, software, resources, formal analysis, and writing – original draft. J.C., M.H., and W.W.: investigation, validation, and data curation. Z.X. and Z.Z.: conceptualization, supervision, funding acquisition, writing – original draft, and review and editing.

## Declaration of interests

The authors declare no competing interests.

## STAR★Methods

### Key resources table

**Table:** 

REAGENT or RESOURCE	SOURCE	IDENTIFIER
**Deposited data**		

25 Carnivoran reference genome assemblies	NCBI RefSeq	See Table S1; https://www.ncbi.nlm.nih.gov/
Mammalia BUSCO v5.4.3 dataset	This paper	See Table S2
Ecological trait dataset for Carnivora	Ding et al.^40^	See Table S3

**Software and algorithms**		

Genome2OR pipeline (OR-gene annotation)	Han et al.^41^	https://doi.org/10.1007/s11427-021-2081-6.
MAFFT (multiple-sequence alignment, E-INS-i)	Katoh & Standley^42^	https://doi.org/10.1093/molbev/mst010.
MEGA 6 (phylogenetic reconstruction, NJ-tree)	Liu et al.^15^	https://doi.org/10.1186/s12864-019-6290-0.
OrthoFinder (orthologous-group inference v2.5.4)	Emms & Kelly^43^	https://doi.org/10.1186/s13059-019-1832-y.
PAML 4.9 (codeml M0 one-ratio model, ω calculation)	Yang^44^	https://doi.org/10.1093/molbev/msm088
R v4.5.0 (statistical analysis, GLM, correlation test)	R Core Team^45^	RRID:SCR_001905
GraphPad Prism v8.0 (plot visualization)	GraphPad Software	RRID:SCR_002798
BUSCO v5.4.3 (genome assembly completeness assessment)	BUSCO Software	RRID:SCR_015008

### Method details

#### Genomic data

In this study, we selected 25 representative Carnivora species based on three criteria for screening and annotating OR genes:

(1) Comprehensive taxonomic coverage, encompassing nine Carnivora families, namely Felidae, Viverridae, Herpestidae, Canidae, Ursidae, Ailuridae, Phocidae, Otariidae and Mustelidae;
(2) Stratified ecological gradients, including 20 terrestrial species with diverse dietary regimes such as omnivores, obligate carnivores, and the bamboo-specialized giant panda, alongside five fully aquatic pinniped species with independent evolutionary origins;
(3) Genomic quality control standards: only publicly available chromosome- or scaffold-level reference genomes with high assembly quality deposited in the NCBI database were selected to ensure the reliability of olfactory receptor gene annotation results.

The full list of species is as follows: leopard, tiger, snow leopard, masked palm civet, asian palm civet, dwarf mongoose, coyote, red fox, giant panda, sun bear, brown bear, asiatic black bear, american black bear, polar bear, spectacled bear, red panda, ringed seal, harbour seal, baikal seal, northern fur seal, steller sea lion, eurasian otter, badger, wolverine, yellow-throated marten (Table S1).

Genome assembly completeness of all 25 carnivoran genomes was assessed using BUSCO v5.4.3 with the mammalia_odb10 dataset containing 14,502 conserved mammalian single-copy marker genes. The proportions of complete single-copy genes, complete duplicated genes, fragmented genes, and missing genes for each assembly were summarized in Table S2.

#### Identification of OR genes

OR genes were identified from genome assemblies using the Genome2OR pipeline.^41^ Genome2OR is an automated pipeline for annotating olfactory receptor genes from chordate genome assemblies, consisting of five integrated modules: nhmmer.py, FindOR.py, IdentifyFunc.py, Batch.py, and Iteration.py.

The annotation process begins with nhmmer.py, which employs clade-specific DNA profile hidden Markov models to scan the genome for OR loci. These profile HMMs were constructed by building DNA multiple sequence alignments (MSAs), under the guidance of protein MSAs, followed by model generation using hmmbuild. Given the substantial sequence divergence of OR coding sequences across chordate evolutionary clades, separate profile HMMs were generated for seven lineages: lancelets, jawless fish, jawed fish, amphibians, reptiles, birds, and mammals.

The nhmmer output was subsequently processed by FindOR.py to extract putative OR coding sequences. For each genomic hit, the fragment was extended to a minimum length of 1,200 bp if shorter, and all potential start and stop codons were identified to define candidate coding regions. Open reading frame (ORF) assessment was performed by scanning for all potential ORFs between start and stop codons in both forward and reverse strands. The longest ORF was selected as the primary candidate, and its length, integrity, and translational continuity were examined. Sequences were preliminarily classified as pseudogenes if the longest potential coding region was shorter than 750 bp, contained frameshift mutations indicated by lengths not divisible by three, or lacked an intact stop codon. Sequences passing these initial checks were designated as potential functional genes and forwarded to IdentifyFunc.py for structural validation. In this module, candidate sequences were merged with template OR sequences and subjected to multiple sequence alignment using MAFFT, followed by segmentation into transmembrane helices, intracellular loops, extracellular loops, and terminal regions. Final classification relied on four criteria: fewer than ten matched conserved patterns, more than ten total amino acid gaps in transmembrane regions, gaps present in more than two transmembrane helices, or abnormal N-terminal length. Sequences failing any criterion were classified as pseudogenes based on their longest intact fragment, whereas those passing all checks were classified as functional genes.

For large-scale applications, Batch.py enabled parallel annotation of multiple genomes, while Iteration.py provided iterative annotation particularly for non-mammalian clades. In this iterative approach, OR sequences identified in the initial round were used to update the profile HMM, which was then applied for subsequent search rounds to capture divergent OR sequences potentially missed by the initial model. The final output comprised two categories: functional genes with intact coding sequences, complete open reading frames, and conserved structural features; and pseudogenes containing frameshift mutations, premature stop codons, or severe structural disruptions.

Compared with the OR gene identification method described by Niimura (2013), the performance of Genome2OR was significantly improved. The Human Olfactory Data Explorer (HORDE) provides state-of-the-art annotations for OR genes, and the results obtained by Genome2OR were highly consistent with HORDE. Specifically, 92.3%–99.4% of the OR sequences identified by Genome2OR in each species could be mapped to HORDE. Notably, the accuracy of Genome2OR consistently outperformed the method proposed by Niimura^26^ for all species analyzed.

#### Phylogenetic tree construction

Protein sequences were aligned with the E-INS-i algorithm implemented in MAFFT using default parameters.^42^ Phylogenetic trees were constructed using the neighbor-joining (NJ) method with Poisson distance in MEGA 6.^15^^,^^16^^,^^19^^,^^46^^,^^47^

#### Orthologous group assignment

We classified all identified intact OR genes using OrthoFinder^43^ and obtained 360 orthologous gene groups (OGGs, orthologous gene clusters). An OGG refers to all descendant genes originating from a single ancestral OR gene shared by the common ancestor of focal species. During mammalian evolution, lineages can only entirely lose an ancestral OGG; gene duplication merely generates extra gene copies within an existing OGG and cannot create brand-new OGG categories. These initial 360 OGGs each harbored at least two gene members. The remaining 103 singleton genes were further sorted via phylogenetic tree analysis. Specifically, we constructed a phylogenetic tree including all 103 singleton OR sequences and non-OR GPCR genes, with non-OR GPCRs set as outgroups. Genes grouped within a monophyletic clade with bootstrap support >70 were assigned to the same OGG, whereas isolated unclustered genes remained as unique singletons.^15^ Following this secondary clustering, the 103 singleton genes were partitioned into an additional 89 OGGs.

Across the 25 carnivoran species examined, all 20,685 intact OR genes were ultimately classified into 374 OGGs and 75 singleton genes. The 374 OGGs each contained two or more intact OR copies, whereas each singleton gene was represented by only a single intact gene across all sampled species. We standardized the naming scheme for all OGGs: Class I OR-derived clusters were prefixed as OGG1, while Class II OR clusters were prefixed as OGG2, followed by sequential numerical labels sorted by the total number of intact gene copies within each OGG.

#### Selection pressure analysis

We calculated the global ω (dN/dS, the ratio of non-synonymous to synonymous substitution rates; this metric quantifies the strength of functional constraints acting on genes, with higher ω values indicating weaker functional constraints) value of each orthologous gene cluster using the M0 one-ratio model in codeml (PAML 4.9). We restricted the analysis to OGGs containing at least three sequences. No site models, branch models, or branch-site models were applied in the present study. The M0 one-ratio model was selected because it generates a single average ω figure representing the overall evolutionary constraint of the entire gene tree of each orthologous cluster, which fits our core aim of comparing mean selection pressure across hundreds of OR gene clusters.^15^^,^^44^

#### Ecological factors comparison in carnivora

To explore the potential link between OR genes and ecological adaptation, we categorized species based on three ecological factors: diet (carnivorous, piscivorous, herbivorous, or omnivorous), habitat (terrestrial or marine), and habitat breadth.^40^ These categories reflect diverse ecological strategies and environmental conditions. Species assignments were based on published data (Figure 5 and Table S3).

### Quantification and statistical analysis

To evaluate ecological differences in olfactory gene repertoires, the Mann–Whitney U test was used to compare intact OR gene numbers and OR pseudogene proportions between marine and terrestrial carnivorans. Boxplots and heatmaps were generated and visualized in GraphPad Prism (v8.0) to illustrate the distribution of the data. The association between intact genes and pseudogenes was assessed using Pearson's product-moment correlation test, implemented in R version 4.5.0.^45^ The correlation coefficient, *p*-value, and R^2^ (coefficient of determination) were computed to evaluate the strength and significance of the linear relationship between the two variables. Generalized linear models (GLMs) were applied to quantify the independent effects of three ecological predictors (habitat type, diet type, and habitat breadth) on three evolutionary traits of OR genes in carnivorans: the count of intact functional OR genes, the proportion of OR pseudogenes, and the evolutionary selection pressure ω (dN/dS ratio). We specified distinct error distributions and link functions for each response variable according to data characteristics:

For the discrete count of intact OR genes, a negative binomial GLM with a log link function was constructed to address potential mild overdispersion in count data^48^; the reference levels for categorical predictors were set as aquatic habitat and specialist diet; For pseudogene proportion (binary count data consisting of pseudogene number and total OR gene number per species), a binomial GLM with a logit link function was adopted to model the odds of OR pseudogenization; For the continuous ω values reflecting selective constraint, a Gaussian GLM with an identity link function was used.

All three full models incorporated habitat type, diet type, and habitat breadth as fixed predictors. We calculated null deviance (intercept-only model) and residual deviance (full model with all predictors), along with the Akaike Information Criterion (AIC), to evaluate overall model goodness-of-fit. Wald z- or t-tests were performed to assess the statistical significance of each regression coefficient. To disentangle the relative explanatory contribution of each ecological predictor, we quantified the reduction in model deviance induced by removing each variable individually, and visualized the deviance reduction values to compare the explanatory power of habitat type, diet type, and habitat breadth across all three OR gene traits.

## Supplemental information

Document S1. Tables S1 and S2

Table S3. Ecological data of 25 species of carnivores

Table S4. Olfactory receptor gene repertoires of 25 carnivoran species
