# Supplementary material for: Similar degenerative patterns of olfactory receptor genes in the giant panda and marine carnivores
Source: iScience. 2026 Oct 17;29(11):117936. doi: 10.1016/j.isci.2026.117936 (PMC13696124; doi:10.1016/j.isci.2026.117936)
Supplement: Supplementary file 1 [file mmc1.pdf]

iScience, Volume *29*

Supplemental information

Similar degenerative patterns of olfactory receptor genes in the giant panda and marine carnivores

Liwen Kang, Junjie Chen, Mingsheng Hong, Wei Wei, Zuofu Xiang, and Zejun Zhang

Table S1. OR number distribution of 25 mammals.

Family	Species	Intact gene number	Pseudogene number	Total number	% Pseudogene	NCBI RefSeq assembly
**Felidae**	Leopard	902	301	1203	25.02%	GCF_024362965.1
**Felidae**	Tiger	892	284	1176	24.15%	GCF_018350195.1
**Felidae**	Snow Leopard	786	337	1123	30.00%	GCF_023721935.1
**Viverridae**	Masked Palm Civet	1393	634	2027	31.28%	GCA_030068075.1
**Viverridae**	Asian Palm Civet	961	414	1375	30.11%	GCA_004024585.1
**Herpestidae**	Dwarf Mongoose	1009	473	1482	31.92%	GCA_004023845.1
**Canidae**	Coyote	765	380	1145	33.19%	GCA_034620425.1
**Canidae**	Red Fox	733	322	1055	30.52%	GCF_003160815.1
**Ursidae**	Giant Panda	702	695	1397	49.75%	GCF_002007445.2
**Ursidae**	Sun Bear	813	536	1349	39.73%	GCA_028533245.1
**Ursidae**	Brown Bear	946	405	1351	29.98%	GCF_023065955.2
**Ursidae**	Asiatic Black Bear	974	456	1430	31.89%	GCA_009660055.1
**Ursidae**	American Black Bear	1276	488	1764	27.66%	GCF_020975775.1
**Ursidae**	Polar Bear	1113	482	1595	30.22%	GCF_017311325.1
**Ursidae**	Spectacled Bear	907	638	1545	41.29%	GCA_028551375.1
**Ailuridae**	Red Panda	793	297	1090	27.25%	GCA_002007465.1
**Phocidae**	Ringed Seal	367	357	724	49.31%	GCA_947044835.1
**Phocidae**	Harbor Seal	340	335	675	49.63%	GCF_004348235.1
**Phocidae**	Baikal Seal	333	318	651	48.85%	GCA_028975605.1
**Otariidae**	Northern Fur Seal	366	545	911	59.82%	GCF_003265705.2
**Otariidae**	Steller Sea Lion	353	459	812	56.53%	GCF_004028035.1
**Mustelidae**	Eurasian Otter	759	554	1313	42.19%	GCF_902655055.1
**Mustelidae**	European Badger	1147	886	2033	43.58%	GCF_922984935.1
**Mustelidae**	Wolverine	990	383	1373	27.89%	GCA_024510155.1
**Mustelidae**	Yellow-throated Marten	1065	450	1515	29.70%	GCA_029410595.1

Table S2. A.) BUSCO evaluation of 25 carnivoran species.

Family	Species	Complete (%)	Single-copy (%)	Duplicated (%)	Fragmented (%)	Missing (%)	Total BUSCO markers
Felidae	Leopard	99	98.1	0.9	0.2	0.8	14502
Felidae	Tiger	98.8	98	0.9	0.3	0.9	14502
Felidae	Snow Leopard	96.9	94.7	2.2	1	2.1	14502
Viverridae	Masked Palm Civet	99	97.2	1.8	0.1	0.8	14502
Viverridae	Asian Palm Civet	76.2	75.5	0.7	8.1	15.7	14502
Herpestidae	Dwarf Mongoose	85.6	84.3	1.3	5.3	9.1	14502
Canidae	Coyote	98.7	97.1	1.6	0.2	1.1	14502
Canidae	Red Fox	96.5	95.5	1.1	1.1	2.3	14502
Ursidae	Giant Panda	96.7	95.4	1.3	1.3	2	14502
Ursidae	Sun Bear	96.2	95.7	0.5	1.2	2.6	14502
Ursidae	Brown Bear	96.8	95.8	1	0.6	2.7	14502
Ursidae	Asiatic Black Bear	99	97.6	1.4	0.4	0.7	14502
Ursidae	American Black Bear	98.2	97.6	0.6	0.5	1.2	14502
Ursidae	Polar Bear	98.4	97.5	0.9	0.5	1.1	14502
Ursidae	Spectacled Bear	98	97.5	0.5	0.6	1.3	14502
Ailuridae	Red Panda	97.4	96.2	1.2	0.8	1.9	14502
Phocidae	Ringed Seal	98.8	97.4	1.4	0.3	0.9	14502
Phocidae	Harbor Seal	98	95.6	2.4	0.6	1.4	14502
Phocidae	Baikal Seal	94.8	93.6	1.2	1.8	3.4	14502
Otariidae	Northern Fur Seal	97.5	95.7	1.7	0.9	1.6	14502
Otariidae	Steller Sea Lion	97.6	95.6	2	0.7	1.7	14502
Mustelidae	Eurasian Otter	98.2	95.4	2.8	0.3	1.5	14502
Mustelidae	European Badger	98.4	95.9	2.5	0.6	1	14502
Mustelidae	Wolverine	99.5	98.7	0.7	0.1	0.4	14502
Mustelidae	Yellow-throated Marten	99.4	98.7	0.6	0.1	0.5	14502

Table S2. B.) Genome assembly quality metrics of 25 carnivoran species.

Family	Species	Genome size	Total ungapped length	Scaffold N50 / L50	Contig N50 / L50	Genome coverage	Sequencing technology
Felidae	Leopard	2.4Gb	2.4Gb	126.8Mb/8	22.2Mb/32	25x	PacBio
Felidae	Tiger	2.4Gb	2.4Gb	146.9Mb/7	74.4Mb/12	78x	PacBio
Felidae	Snow Leopard	2.4Gb	2.4Gb	112.1Mb/9	179.5/kb/3996	31.94x	Illumina
Viverridae	Masked Palm Civet	2.4Gb	2.4Gb	111.8Mb/9	12.9Mb/58	216x	PacBio
Viverridae	Asian Palm Civet	2.5Gb	2.5Gb	71.8kb/8704	62.9kb/10106	36.9x	Illumina
Herpestidae	Dwarf Mongoose	2.4Gb	2.4Gb	179.1kb/3602	113.6kb/5829	32.3x	Illumina
Canidae	Coyote	2.3Gb	2.3Gb	64.5Mb/14	42Mb/19	48x	PacBio
Canidae	Red Fox	2.4Gb	2.4Gb	12.5Mb/55	55.5kb/12033	93.9x	Illumina
Ursidae	Giant Panda	2.4Gb	2.4Gb	129.2Mb/8	127.7kb/5332	93x	Illumina
Ursidae	Sun Bear	2.5Gb	2.5Gb	59.7Mb/16	73.7kb/9389	105x	Illumina
Ursidae	Brown Bear	2.4Gb	2.4Gb	72Mb/14	35.7Mb/22	32x	PacBio
Ursidae	Asiatic Black Bear	2.4Gb	2.4Gb	26.8Mb/27	146kb/4841	90.61x	Illumina
Ursidae	American Black Bear	2.4Gb	2.4Gb	13.9Mb/43	13.9Mb/43	11x	PacBio
Ursidae	Polar Bear	2.3Gb	2.3Gb	72.2Mb/13	206.7kb/3292	52x	Illumina
Ursidae	Spectacled Bear	2.3Gb	2.3Gb	104.7Mb/9	117.3kb/5804	147x	Illumina
Ailuridae	Red Panda	2.3Gb	2.3Gb	3Mb/215	99.6kb/6772	115.5x	Illumina

Phocidae	Ringed Seal	2.4Gb	2.3Gb	21Mb/30	154.8kb/4532	150x	Illumina
Phocidae	Harbor Seal	2.4Gb	2.3Gb	41Mb/18	283.9kb/2463	45x	Illumina
Phocidae	Baikal Seal	2.3Gb	2.2Gb	151.5Mb/7	14.3kb/46423	110x	Illumina
Otariidae	Northern Fur Seal	2.4Gb	2.4Gb	47Mb/14	161kb/4372	40x	Illumina
Otariidae	Steller Sea Lion	2.4Gb	2.4Gb	14Mb/54	242.4kb/2995	34.65x	Illumina
Mustelidae	Eurasian Otter	2.4Gb	2.4Gb	149Mb/7	30.4Mb/23	63x	PacBio
**Mustelidae**	European Badger	2.7Gb	2.7Gb	133Mb/8	75.2Mb/12	85x	Illumina
**Mustelidae**	Wolverine	2.4Gb	2.4Gb	144Mb/7	36.6Mb/20	96.87x	Illumina
**Mustelidae**	Yellow-throated Marten	2.4Gb	2.4Gb	143.1Mb/7	68.6Mb/12	107.96x	PromrthION
